# Covalently Functionalized Carbon Nano-Onions Integrated Gelatin Methacryloyl Nanocomposite Hydrogel Containing γ-Cyclodextrin as Drug Carrier for High-Performance pH-Triggered Drug Release

**DOI:** 10.3390/ph14040291

**Published:** 2021-03-25

**Authors:** Narsimha Mamidi, Ramiro Manuel Velasco Delgadillo, Enrique V. Barrera

**Affiliations:** 1 Department of Chemistry and Nanotechnology, School of Engineering and Science, Tecnologico de Monterrey, Monterrey, Nuevo Leon-64849, Mexico; ramirov2607@gmail.com; 2 Department of Materials Science and Nanoengineering, Rice University, Houston, TX 77005, USA; ebarrera@rice.edu

**Keywords:** GelMA/f-CNOs/CD supramolecular hydrogels, stimuli-responsive drug release, mechanical properties, cell viability

## Abstract

Herein, poly (*n*-(4-aminophenyl) methacrylamide)) carbon nano-onions (PAPMA-CNOs = f-CNOs) and γ-cyclodextrin/DOX-complex (CD) reinforced gelatin methacryloyl (GelMA)/f-CNOs/CD supramolecular hydrogel interfaces were fabricated using the photo-crosslinking technique. The physicochemical properties, morphology, biodegradation, and swelling properties of hydrogels were investigated. The composite hydrogels demonstrated enriched drug release under the acidic conditions (pH 4.5 = 99%, and pH 6.0 = 82%) over 18 days. Owing to the f-CNOs inclusion, GelMA/f-CNOs/CD supramolecular hydrogels presented augmented tensile strength (σ_ult_ = 356.1 ± 3.4 MPa), toughness (K = 51.5 ± 0.24 Jg^−1^), and Young’s modulus (E = 41.8 ± 1.4 GPa). The strengthening of GelMA/f-CNOs/CD hydrogel systems indicates its good dispersion and the degree of polymer enveloping of f-CNOs within GelMA matrixes. Furthermore, the obtained hydrogels showed improved cell viability with human fibroblast cells. Nevertheless, the primed supramolecular hydrogels would pave the way for the controlled delivery systems for future drug delivery.

## 1. Introduction

Natural or synthetic hydrogels are absorbent macromolecular network systems capable of aquatic absorption. Due to their three-dimensional porous network, flexibility, and high water content, hydrogels are attractive biomaterial scaffolds for drug delivery, [1,2,3] tissue engineering, [4] vaccines, [5,6] and biosensors [7]. Several crosslinking strategies including chemical conjugations, [8,9] physical interactions, [10,11] and polymerizations [12,13] have been formulated for hydrogel synthesis. However, the fabrication of next-generation hydrogels with amended functional and structural recapitulations of damaged organs remains a challenge. In this context, supramolecular hydrogels may offer access to innovative material characteristics that are inaccessible based on conventional principles [14]. The benefits of supramolecular hydrogels comprise control over the physicochemical and structural properties in a dynamic, reversible, and biomimetic manner. Under specific stimuli conditions, such as the light, [15,16,17] temperature, [18,19] pH, [20,21,22] and redox, [22,23] the supramolecular hydrogel systems instigate their motions and structures to release encapsulated cargos in the targeted site. 

Among these stimuli, pH is particularly attractive and pH-responsive supramolecular hydrogels have ascended as impending biomaterial conjugates in numerous applications [20,21,22]. Hydrogels have inimitable benefits and can act as drug vehicles for a prolonged and controlled release of therapeutic agents, proteins, and other macromolecules. Specifically, hydrogels prepared using naturally occurring biopolymers have promising advantages over synthetic hydrogels, including low immune response, excellent biocompatibility, and functionalization in their chemical structures [24] For instance, gelatin/gelatin methacryloyl (GelMA), [25,26] zein protein, [27,28] chitosan, [29] and hyaluronic acid [30] -based hydrogels have been fabricated for biomedical applications. Among these biopolymers, GelMA is widely used in drug delivery, tissue engineering, and regenerative medicine [25,31]. However, rapid degradation, poor water resistance, and weak mechanical properties of gelatin are impeding its broad applications in various research fields. 

Therefore, carbon nanotubes (CNTs), graphene oxide (GO), and carbon nano-onions (CNOs) were integrated into gelatin to improve the mechanical and biological properties of gelatin-based hydrogels [26,32,33]. Among these carbonaceous materials, CNOs are considered the most promising carbon materials for biomedical applications due to their high surface area, negligible toxicity, and biocompatibility [34]. Besides this, CNOs exhibited inferior toxicity and low inflammatory nature compared to CNTs [34]. Moreover, surface-functionalized CNOs embedded composite scaffolds showed enhanced mechanical, thermal, and controlled drug delivery properties [26,28,35,36,37,38]. Recently, poly (*N*-(4-aminophenyl) methacrylamide))-carbon nano-onions (PAPMA-CNOs = f-CNOs) were synthesized and reinforced into anilinated-poly (ether ether ketone) (AN-PEEK) to fabricate AN-PEEK/f-CNOs nanocomposite thin films. The obtained thin films presented augmented mechanical properties, cell viability, and pH-responsive drug release [39]. These remarkable outcomes of f-CNOs incited us to develop f-CNOs reinforced GelMA hydrogels. 

Cyclodextrins (CDs) are biocompatible cyclic oligosaccharides and prepared by bacterial digestion of cellulose [40]. Typical CDs are classified as α-CD, β-CD, and γ-CD macromolecules. Among them, γ-CD was used as a potential stabilizer and solubilizing agent in food products and pharmaceuticals [41]. The γ-CD possesses most promising toxicological profile, highest water solubility, and largest hydrophobic cavity compared to α-CD and β-CD [41]. Due to the above multi favorable characteristics, pristine γ-CD and γ-CD incorporated several biomaterial were used as promising drug carriers for doxorubicin (DOX) [40,41,42]. DOX is one of the best prevailing anticancer drugs, widely utilized in the treatment of leukemia and several solid tumors. However, DOX shows some drawbacks, including poor water solubility, emerging multidrug resistance, and severe cardiotoxicity [43]. To curtail these problems, suitable drug-loading strategies have been developed. For instance, PEGylated liposomal DOX nanoparticles have formulated and marketed [44].

In the present study, DOX encapsulated DOX/γ-CD inclusion complex and f-CNOs were reinforced into GelMA to fabricate supramolecular hydrogels. To the best of our knowledge, the fabrication of DOX-loaded GelMA/f-CNOs/CD nanocomposite hydrogel has not been reported. We hypothesized that γ-CD can easily form the host-guest interactions with DOX due to its hydrophobic cavity, which can efficiently release DOX under stimuli conditions, whereas f-CNOs can enhance the mechanical properties of hydrogels. GelMA was selected as the building block owing to its biocompatibility as a naturally attaining biopolymer. Besides this, GelMA is a key constituent of an extracellular matrix and it can present improved biodegradability, swelling, and cell viability. Accordingly, DOX-loaded GelMA/f-CNOs/CD hydrogels were primed for the investigation of stimuli-responsive drug release. The obtained hydrogels showed improved cell viability against human fibroblast cells over three days of cultivation. Owing to CD incorporation, supramolecular hydrogels presented pH-responsive sustained drug release over 18 days. Due to f-CNOs inclusion, the supramolecular hydrogels presented enhanced tensile strength, toughness, and Young’s Modulus.

## 2. Results and Discussion

Initially, f-CNOs were synthesized using a previous report and directly used for further studies [39]. The hydrodynamic size distribution, N_2_ gas adsorption/desorption isotherm, and pore size dissemination of f-CNOs were studied (Figure S1). The f-CNOs possessed very good hydrodynamic size, porosity, and surface area. Citric acid cross-linked γ-cyclodextrin was prepared according to the previous procedure and characterized by using FTIR analysis (Scheme S1) [40]. Besides this, we studied the drug loading efficiency of γ-cyclodextrin. We found that the amount of DOX bound in a cavity of γ-cyclodextrin was pH-dependent (Figure S2a). This could be due to the increased hydrophilicity and solubility of DOX at acidic pH caused by protonation of –NH_2_ group on DOX, thereby diminishing the electrostatic interactions between γ-cyclodextrin and DOX molecules. In terms of drug release, we found that DOX loaded in a cavity of γ-cyclodextrin endured stably bound in basic solution at room temperature (Figure S2b). Under pH 4.5, we measured considerable DOX release from γ-cyclodextrin by ~42% over 4 days (Figure S2b), attributed to the enhanced solubility and hydrophilicity of DOX at this pH. In addition, we measure the stability of DOX under different pH solutions (pH 4.5–9.5) and found good stability of DOX in acidic and alkaline solutions (Figure S3). Furthermore, the formation DOX/γ-cyclodextrin inclusion complex (DOX@γ-CD IC) was confirmed by FTIR, DSC, TGA, fluorescence, and Benesi-Hildebrand equation (Figure S4, Supplementary Materials). The results demonstrated that DOX molecules were successfully encapsulated in the γ-cyclodextrin cavity and formed the DOX@γ-CD IC (Figure S4). Thus, the good stability of DOX@γ-CD IC (hereafter CD) under basic solutions and controlled release under acidic pH solutions are promising features for advanced drug delivery technologies. 

**Scheme 1:**
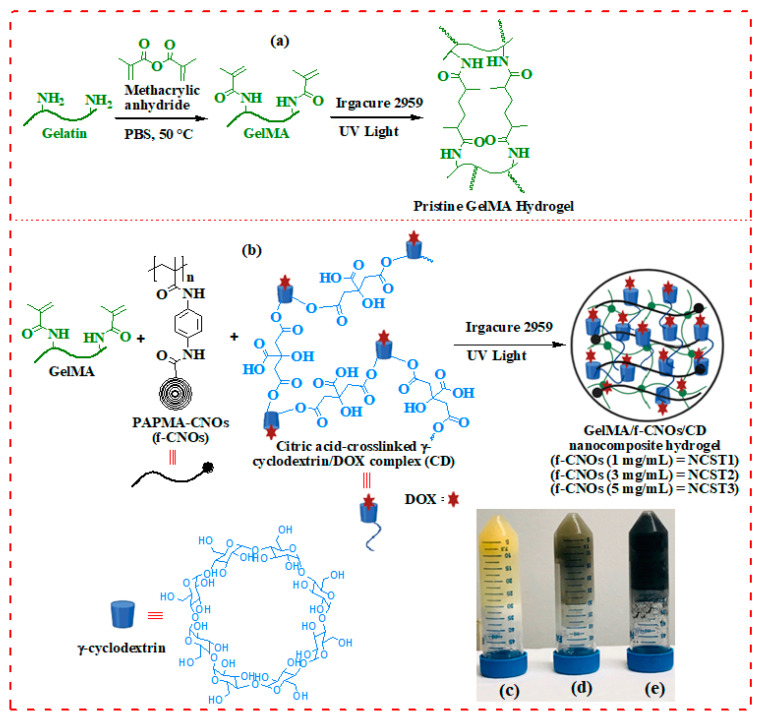
(**a**) Synthetic route of GelMA from gelatin and methacrylic anhydride (MA). (**b**) A schematic to define the formulation of GelMA/f-CNOs/CD hydrogels. GelMA was covalently cross-linked using visible light in the presence of Irgacure 2959 as a photoinitiator. Digital Photographs of fabricated hydrogels (**c**) pristine GelMA, (**d**) NCST1, and (**e**) NCST3 hydrogels, respectively. Abbreviation: NCST, Nanocomposite hydrogel.

The synthesis of GelMA and fabrication of pristine GelMA hydrogel were illustrated in Scheme 1a. Initially, GelMA was synthesized from the coupling reaction of gelatin with MA in PBS at 50 °C followed by freeze-drying to attain GelMA (Scheme 1a). This reaction step introduces methacryloyl groups on gelatin.

**Figure 1:**
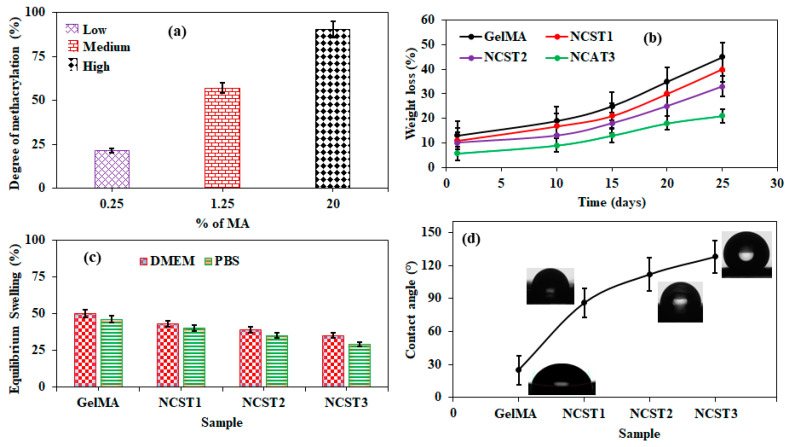
(**a**) Degree of methacrylation as determined by TNBS study; three-volume percentages (0.25%, 1.25%, and 20%) of MA were explored to scrutinize the degree of methacrylation of GelMA. (**b**) Degradation, (**c**) swelling, and (**d**) water contact angle of pristine GelMA and NCST hydrogels under 600 s UV exposure.

The degree of methacrylation of gelatin was determined using the Habeeb method [45]. These results revealed the aptitude to generate GelMA macromers with a degree of methacrylation varying approximately from 23% to 92%. Three sets of GelMA were generated with low (23.9 ± 1.7%), medium (56.4 ± 8.3%), and high (92.1 ± 2.5%) degree of methacrylation corresponding to 0.25%, 1.25%, and 20% (*v/v*) of MA (Figure 1a). Thus, a high (92.1 ± 2.5%) degree of methacrylation based GelMA was used for further studies. Initially, pristine GelMA hydrogels were fabricated by using 10% of GelMA and 0.5% of Irgacure 2959 under UV irradiation (photo-crosslinking). During the hydrogel fabrication, GelMA solution was exposed to UV irradiation for 1 to 10 min to obtain GelMA hydrogel with different crosslinking densities. Under the same optimized condition, GelMA composite hydrogels were prepared by integrating CD (5% *w/v*), and f-CNOs with different concentrations (1, 3, and 5 mg/mL). Similarly, GelMA/f-CNOs/CD composite hydrogels were prepared with different crosslinking densities by varying UV exposure times (1 to 10 min). The obtained GelMA/f-CNOs/CD hydrogels with 1, 3, and 5 mg/mL of f-CNOs indicated as NCST1, NCST2, and NCST3 nanocomposite hydrogels, respectively (Scheme 1b). The digital photographs of pristine GelMA, NCST1, and NCST3 hydrogels were illustrated in Scheme 1c–d, respectively. The inverted images reveal the confirmation of the gelation. 

The degradation characteristics of hydrogels play a crucial role in the construction of a cellular microenvironment suitable for tissue engineering and drug delivery; for instance, GelMA comprises aboard sequences of matrix metalloproteinases (MMPs) [25,31].Consequently, cells encapsulated into GelMA hydrogels can destroy and amend the nearby hydrogel by replacing it with cell-secreted extracellular matrix (ECM) of native cells [25,31].Therefore, degradation of GelMA and NCST hydrogels were assessed with a function of UV exposure times (100, 250, and 500 sec), and the results are presented in Figure S5. As showed in Figure S5, UV exposure time (crosslinking density) obviously affected the degradation of hydrogels when subjected to a degradation test over 48 h. Initially, neat GelMA and NCST hydrogels exhibited a very high degradation rate under 100 sec UV exposure. However, the degradation rate of the specimens was gradually decreased with increasing the crosslinking density (250 and 500 sec UV exposure). Thus, Figure S5a reveals that degradation profile of NCST1, NCST2, and NCST3 hydrogels are slightly similar to that of neat GelMA hydrogel alone. Furthermore, biodegradation of pristine GelMA and NCST hydrogels was studied at even longer UV exposure time (600 sec), and the results are presented in Figure 1b. Pristine GelMA hydrogel showed around 45% of degradation over 25 days of incubation (Figure 1b). This could be due to the presence of unprotected hydrophilic amino acid residues and porosity of the hydrogel. In contrast, NCST hydrogels exhibited controlled and sustained degradation throughout the study (Figure 1b). Specifically, NCST1 hydrogels revealed approximately 38% of degradation over 25 days, whereas NCST2 hydrogel displayed around 33% of degradation. On the other hand, NCST3 hydrogel showed the lowest degradation (~21%) throughout the experiment, which could be due to the higher content of f-CNOs and stronger electrostatic and π-π interactions among GelMA, CD, and f-CNOs functional groups. Besides, the preservation of f-CNOs structure after integration with GelMA and CD was demonstrated by Raman spectra (Figure S7).

The swelling properties of a hydrogel network are important in several applications as they influence solute diffusion, mechanical properties, surface properties, and mobility. The swelling behavior of hydrogels is reliant on the interaction between solvent and polymer, cross-linking density, and the porosity of the hydrogel network.^31^ Initially, we measure the swelling performance of hydrogels with the functional of crosslinking density under DMEM (pH 7.4) at 37 °C (Figure S5b). We found that at lower UV exposure time (100 sec), all the hydrogels exhibited a wide range of swelling properties. Approximately 1.700%, 1.633%, 1.487%, and 1.395% of swelling ratios were observed from pristine GelMA, NCST1, NCST2, and NCST3 hydrogels under UV exposure time of 100 sec, respectively (Figure S5b). The swelling ratios were progressively decreased by increasing UV exposure times. After 500 sec of crosslinking, pristine GelMA, NCST1, NCST2, and NCST3 hydrogels showed around 267%, 156%, 114%, and 105% of swelling ratios, respectively (Figure S5b). These results demonstrate that the swelling ratios of hydrogels could be tunable by crosslinking density and the concentration of f-CNOs. Inspired by these results, we further investigated the swelling behavior of hydrogels under physiological conditions (PBS and DMEM) at longer UV exposure time (Figure 1c). Pure GelMA hydrogel exhibited around 50% and 46% swelling in DMEM and PBS solutions, respectively (Figure 1c). NCST1 hydrogels showed 43% and 40% swelling in DMEM and PBS, respectively, whereas, NCST2 hydrogel displayed approximately 39% and 35% swelling in DMEM and PBS, respectively. NCST3 composite hydrogels presented 34% and 29% swelling in DMEM and PBS, respectively. Overall, the swelling behavior of hydrogels was slightly lower in PBS compared to those in DMEM. The results demonstrated that crosslinking density, and content of f-CNOs, have a detrimental effect on the swelling.

The wettability of hydrogels can stimuli cell adhesion and proliferation that would influence drug delivery and tissue engineering applications. Therefore, the wettability of hydrogels was measured, and the results were illustrated in Figure 1d. Neat GelMA hydrogel showed around 25 ± 1° of contact angle. NCST1 hydrogel presented approximately 86 ± 2°, and NCST2 hydrogel exhibited 112 ± 1° of water contact angles, whereas, NCST3 hydrogel indicated 128 ± 1° of water contact angle. The wettability was dependent on the content of f-CNOs, and the lowest wettability was observed at the higher concentration of f-CNOs (NCST3 with 5 mg/mL). Thus, the wettability results demonstrated that the hydrophobic nature of NCST hydrogels was significantly improved. This could be due to the hydrophobic behavior of f-CNOs and supramolecular interactions among CD, f-CNOs, and GelMA macromolecules.

**Figure 2:**
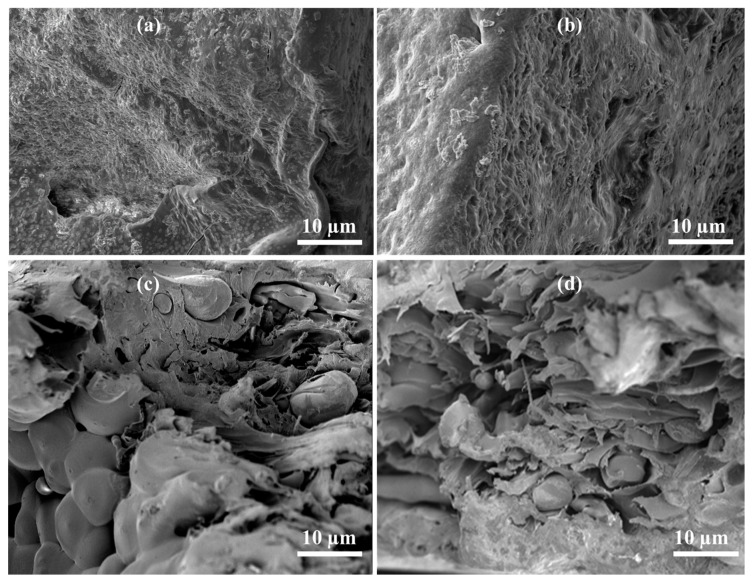
The morphology of the hydrogel interior of (**a**) pristine GelMA, (**b**) NCST1, (**c**) NCST2, and (**d**) NCST3, respectively.

Besides this, the porosity of hydrogels with the function of crosslinking densities was also measured, and good porosity was observed on the surface of GelMA and NCST hydrogels (Figure S5c). As shown in Figure S5c, pristine GelMA and NCST hydrogels exhibited good pore distribution under UV exposure times of 100, 250, and 500 sec. Specifically, pristine GelMA hydrogel displayed around 7.7 ± 1.3 and 4.3 ± 1.7 µm of pore sizes under UV exposure time of 100 and 500 sec, respectively. In contrast, with the incorporation of f-CNOs and increasing crosslinking density, the pore size of NCST hydrogels was slightly increased at all UV exposure times. Overall, the porosity of pristine GelMA hydrogel was slightly lower than that of NCST1, NCST2, and NCST3 hydrogels. This could be due to the high number of MA groups on GelMA are cross-linked under UV light, on the other hand with the incorporation of f-CNOs into GelMA, the MA groups of GelMA might have involved in electrostatic interactions with f-CNOs networks, which shielded MA groups of GelMA from UV light. Next, we also measured the porosity and morphology of hydrogels under longer UV exposure time (600 sec) to investigate the influence of crosslinking density on the surface morphology of hydrogels (Figure 2). Because surface morphology of a drug vehicle is one of the most essential features of the prevailing drug release platform. The surface morphology of the hydrogels was investigated by using Scanning electron microscopy (SEM). SEM images of pristine GelMA and NCST hydrogels with different contents of f-CNOs (1, 3, and 5 mg/mL) were presented in Figure 2a–d. NCST2 and NCST3 hydrogel samples showed a rough and crumple structure with improved porosity (Figure 2c,d). On the other hand, neat GelMA and NCST1 hydrogels showed sponge-like structures with lower porosity (Figure 2a,b). As can be seen from Figure 2b–d, with increasing f-CNOs content, the surface roughness and porosity of NCST hydrogels were gradually increased. This highly porous morphology of NCST2 and NCST3 hydrogels can restore the water uptake characteristics. This can be attributed to the effect of intercalated f-CNOs and CD, which makes electrostatic, supramolecular π-π stacking and hydrogen bonding interactions with functional groups of GelMA. 

**Figure 3:**
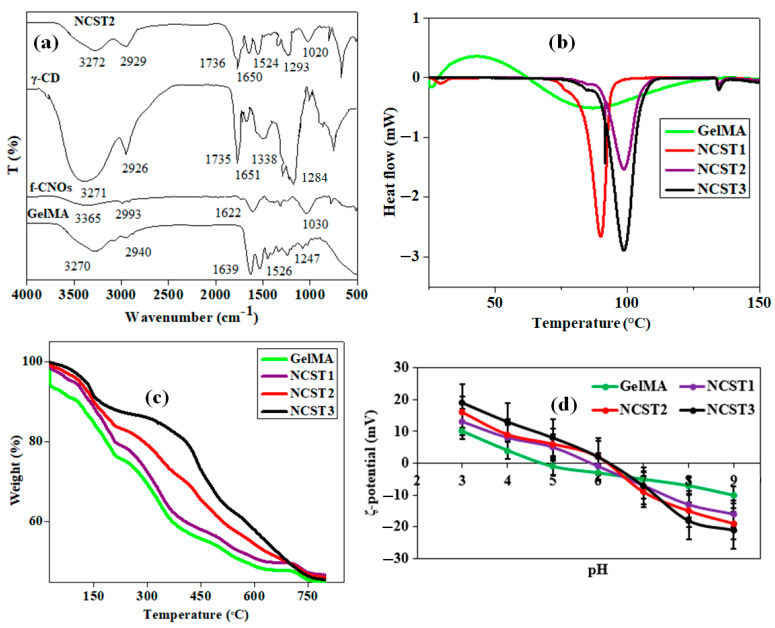
(**a**) FTIR, (**b**) DSC, (**c**) TGA, and (**d**) ζ-potential curves of pristine GelMA and NCST hydrogels, respectively.

FTIR analysis could present enough information to determine the possible interactions among GelMA, f-CNOs, and γ-cyclodextrin. Therefore, FTIR spectra of neat GelMA, f-CNOs, γ-cyclodextrin, and composite hydrogels were measured and illustrated in Figure 3a. As shown in Figure 3a, neat GelMA showed five significant absorption bands at 3270, 2940, 1639, 1526, and 1247 cm^−1^, which could be correspondingly denoted to the stretching frequencies of N-H, C-H, C=O of amide I and II, and N-H bending and C-N stretching vibrations of gelatin backbone. The FTIR spectra of f-CNOs displayed three major characteristic bands approximately at 3365, 2993, and 1622 cm^−1^ for N-H, C-H stretching, and C=O of amide (CONH), respectively (Figure 3a). 

On the other hand, citric acid cross-linked γ-cyclodextrin showed a characteristic peak at 1735 cm^−1^ for C=O stretching of ester groups. This revealed the esterification reaction was successful between citric acid and cyclodextrin. The other peaks at 3271, 2926, 1284, and 1018 cm^−1^ assigned to O-H stretching, C-H stretching, C-O-C, and C-O stretching vibrations of cyclodextrin, indicate the essential chemical structure of cyclodextrin preserved. After the formulation of nanocomposite hydrogels, the absorption bands of NCST2 hydrogel shifted to slightly higher frequencies. Specifically, NCST2 hydrogel exhibited broad new peaks approximately at 3272 cm^−1^ for N-H/O-H stretching, 2929 for C-H stretching, and 1736 cm^−1^ for C=O stretching of the ester groups. Besides this, C-O-C and C-O stretching vibrations of NCST2 hydrogel were also detected in the different frequencies. Due to the existence of an overlap between the guest and host macromolecules, not all the changes of FTIR shifts can be noticed in the hydrogels. The FTIR spectrum of NCST1, NCST2, and NCST3 hydrogels are very similar; thus, we have shown only FTIR spectrum for NCST2 hydrogels in the Figure 3a. Accordingly, FTIR results revealed the electrostatic, π-π stacking, and hydrogen bonding interactions among GelMA, f-CNOs, and CD macromolecule.

The melting temperature (T_m_) of specimens was measured by using DSC analysis and DSC thermograms of GelMA, and NCST hydrogels were illustrated in Figure 3b. The melting temperatures of the hydrogels observed in a range of 90–111 °C. Pristine GelMA hydrogel showed around 92.0 °C of T_m_, whereas 98.0, 103.0, 106.0 °C of T_m_ were observed from NCST1, NCST2, and NCST3 hydrogels, respectively. Compared with pure GelMA, NCST hydrogels displayed completely different and a sharp DSC peak pattern. This could be due to the Van der Waals forces, π-π stacking, and hydrogen bonding interactions between f-CNOs and GelMA. Thus, melting temperature of composites hydrogels were significantly enhanced with the incorporation of f-CNOs.

The TGA thermograms of hydrogels were depicted in Figure 3c. The thermal degradation behavior of GelMA hydrogel can be observed in Figure 3c. All the hydrogel specimens exhibit a minor loss of mass in a temperature range of 75–145 °C, perhaps instigated by the removal of entrapped water within hydrogel specimens. Besides this, the hydrogels showed thermal decomposition in a range of 195–468 °C. Thus, TGA results validated that the thermals stability of NCST hydrogels was significantly improved with the inclusion of f-CNOs. This could be due to the high thermal conductivity behavior of CNOs and electrostatic interactions between GelMA and f-CNOs. 

The zeta-potential analysis was performed to investigate the surface charge behavior of hydrogels under different pH solutions (pH 3–9), and the results were depicted in Figure 3d. All the hydrogel samples displayed positive ζ-potential values under acidic buffer solutions, whereas negative ζ-potential values were observed under basic buffer solutions (Figure 3d). Specifically, pristine GelMA showed +9.3 ± 0.45 and −10.1 ± 1.23 mV of ζ-potential in pH 3.0 and pH 9.0, respectively. NCST1 hydrogel presented +13.0 ± 0.23, and −16.6 ± 0.78 mV of ζ-potential values in pH 3.0 and pH 9.0, respectively. NCST2 hydrogel exhibited around +16.3 ± 1.13, and −19.1 ± 1.18 mV of ζ-potential values in pH 3.0 and pH 9.0, respectively. Meanwhile, NCST3 hydrogel unveiled around +19.2 ± 0.54, and −21.7 ± 0.69 mV of ζ-potential values in pH 3.0 and pH 9.0, respectively. Overall, zeta potential results demonstrated that all the hydrogels presented positive ζ-potential values under acidic buffer, it attributed that hydrophilic functional groups of polymers are protonated. On the other hand, under basic conditions, all the hydrophilic groups of hydrogels might have deprotonated and showed negative ζ-potential values. 

The tolerance of high loads, as well as sustained deformation of the hydrogels, are necessary characteristics in drug delivery and tissue engineering applications. However, it is known that the degree of crosslinking density significantly influences the mechanical characteristics of GelMA-based hydrogels [25]. For this, the tensile stress of GelMA, and NCST hydrogels were vetted with a function of UV exposure times (100 to 500 sec), and the results were presented in Figure S5d. Pristine GelMA showed tensile stress in a range of 10.0 ± 1.1 to 143.1 ± 1.7 MPa with increasing UV curing time (100 to 500 sec). Thus, at a longer UV exposure time (500 sec), neat GelMA hydrogel displayed enhanced tensile stress due to the MA groups present in the GelMA gel were almost cross-linked. On the other hand, NCST hydrogels exhibited a wider range of tensile stresses (21.2 ± 2.1 to 297.1 ± 1.9 MPa) because of the strong electrostatic interactions between f-CNOs, and MA groups on GelMA chains. Within this range, the tensile stress also gradually augmented with increasing f-CNOs contents (1 to 5 mg/mL) and UV exposure times (100 to 500 sec). These results suggesting that the rigid reinforcement induced by f-CNOs may have improved the tensile stress of composite hydrogels. 

**Figure 4:**
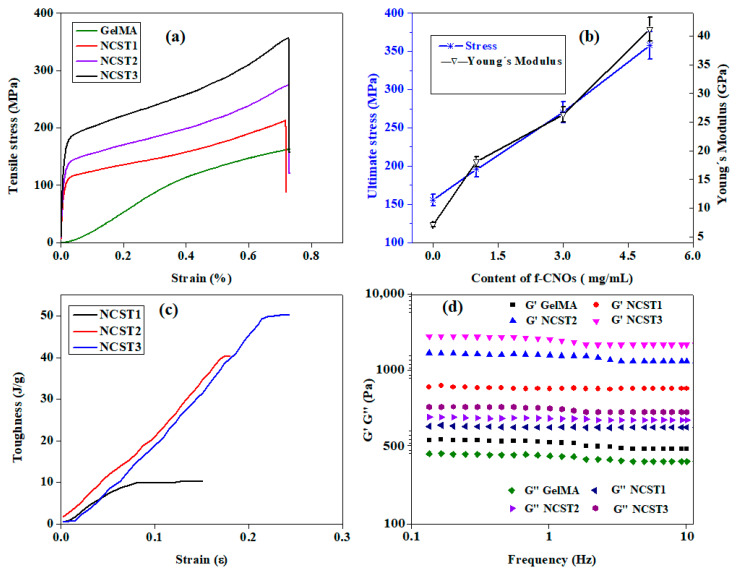
The curves illustrating the mechanical properties of hydrogels: (**a**) tensile stress, (**b**) tensile strain vs. Young’s modulus, (**c**) toughness, and (**d**) storage and loss modulus (G’ G’’).

Provoked by these outstanding results, we have also measured full-fledged mechanical properties including non-linear stress/strain curve, Young’s modulus, and the toughness of hydrogels under longer UV exposure time (600 sec) (Figure 4a–c). Improved mechanical properties were observed with the inclusion of f-CNOs within GelMA matrixes of hydrogels at longer UV exposure time (600 sec). Specifically, 356.12 ± 3.67, 274.24 ± 5.71, and 212.95 ± 1.71 MPa of the tensile strength (σ) were observed from NCST3, NCST2, and NCST1 hydrogels, respectively (Figure 4a). On the other hand, pristine GelMA hydrogel showed 157.81 ± 3.91 MPa of tensile strength. In addition, NCST3 hydrogel exhibited Young’s modulus (E) of 41.19 ± 3.78 GPa, whereas NCST2 hydrogels showed E of 26.32 ± 1.09 GPa, and NCST1 hydrogel displayed around 18.12 ± 4.71 GPa of E (Figure 4b). Pure GelMA hydrogel presented around 7.12 ± 3.1 GPa of E. Thus, with the inclusion of f-CNOs, 2.5, 3.7, and 5.8 times of E was improved in NCST1, NCST2, and NCST3 hydrogels, respectively (Figure 4b). Moreover, the toughness of pristine GelMA and NCST hydrogel specimens were studied and illustrated in Figure 4c. The toughness of specimens measured to be 50.24, 40.32, and 10.31 Jg^−1^ for NCST3, NCST2, and NCST1 hydrogels, respectively (Figure 4c). The toughness measurements of composite hydrogels revealed the substantial effect of concurrent changes in the concentration of f-CNOs, and stress transfer conditions at the interface between f-CNOs and GelMA. 

Therefore, the changes in toughness and tensile strain among NCST hydrogels can be validated for their different abilities to restructure under tensile stress. Nevertheless, the NCST hydrogels displayed augmented mechanical properties as compared to the pristine GelMA hydrogel under high crosslinking density. This could be due to the high crosslinking density, π-π stacking, electrostatic interfaces, and strong hydrogen bonding interactions between f-CNOs and GelMA backbone. To further confirm the growth in stiffness provided by the incorporation of f-CNOs and crosslinking density in the GelMA networks, we measured the storage modulus (G’) of GelMA and NCST hydrogels as a function of frequency (Figure 4d). The G’ values of all the composite hydrogels were almost independent of the frequency applied, which evidenced that all the composite hydrogels displayed a solid-like behavior. Particularly, NCST3 with the highest content of f-CNOs showed the highest G′ values among the tested hydrogels. Besides this, all composite hydrogels displayed higher storage modulus (G′) values than their loss modulus (G″) values, indicating that elastic behavior is dominated when a load was applied. Similar results were observed from the nanodiamond-reinforced GelMA hydrogels [46]. We also measured the degree of crosslinking (DC) of hydrogels with a function of UV exposure times (Figure S6). We found around 76%, 72%, 70%, and 69% of DC from pristine GelMA, NCST1, NCST2, and NCST3 hydrogels, respectively, over 600 sec of UV exposure time (Figure S6).

**Figure 5:**
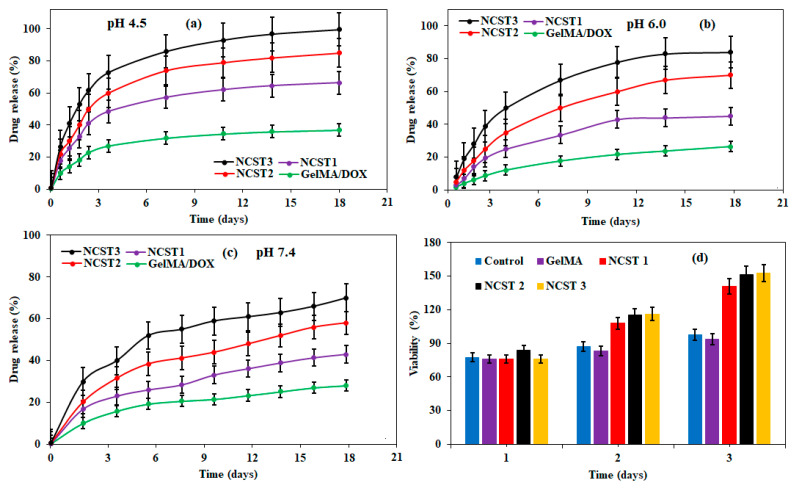
Stimuli-responsive drug release of DOX loaded GelMA, NCST1, NCST2, and NCST3 hydrogels at (**a**) pH 4.5, (**b**) pH 6.0, and (**c**) pH 7.4, respectively. (**d**) Cell viability of hydrogels with human fibroblast cells.

The structural and mechanical properties of hydrogels are essential characteristics to mimic the structures of ECM [31]. It is known that the pH of the microenvironment of cancer cells (pH 4.5−6.5) [47] is different from the pH of normal cells (pH 7.4) [48]. To study whether the current hydrogels are suitable for pH values of the microenvironment of tumors and normal cells, we have vetted the in vitro drug release from composite hydrogels under pH 4.5, 6.0, and 7.4 (Figure 5a–c). DOX-loaded pristine GelMA hydrogel showed around 37.5% of drug release for over 18 days of study at pH 4.5, whereas NCST1 hydrogels displayed approximately 66.4% of release under the same conditions (Figure 5a). Besides, NCST2 and NCST3 hydrogels exhibited 85% and 99.6% of persistent drug release at pH 4.5 over 18 days of incubation, respectively (Figure 5a). These drug release results revealed that the drug release behavior is considerably reliant on the pH environment of the solution. Next, drug release measurements were conducted under pH 6.0 to explore the drug release behavior of hydrogels. As shown in Figure 5b, DOX-loaded GelMA, NCST1, NCST2, and NCST3 hydrogels displayed 26.5%, 45.4%, 70.2%, and 82.1% of drug release, respectively. Under physiological conditions (pH 7.4), DOX-loaded pure GelMA hydrogel showed around 23.3% of drug release over 18 days. 

On the other hand, NCST1, NCST2, and NCST3 hydrogels exhibited around 41.9%, 56.1%, and 66.9% of drug release, respectively (Figure 5c). It is attributed that DOX molecules are strongly bound in the hydrophobic cavity of γ-cyclodextrin under a physiological environment. Thus, composite hydrogels presented a higher drug release profile at pH 4.5 compared to pH 6.0 and 7.4 (Figure 5a–c). The drug release measurements revealed that the solubility and hydrophilicity of DOX molecules might have enhanced under pH 4.5 due to the protonation of the glycosidic amine group of DOX. At pH 4.5, the protonation of DOX resulted in the destruction of GelMA/f-CNOs/CD hydrogel, which facilitated the dissociation of γ-cyclodextrin/DOX complex and more drug release. There was a slight burst release at the beginning of the drug release profile at the acidic environment, perhaps because the DOX molecules inclined to move to the surface of cyclodextrin due to their hydrophilic nature. Nevertheless, NCST hydrogels were exceptionally sensitive at pH 4.5 and exhibited maximum drug release (99.6%, Figure 5a).

The cytotoxicity of hydrogels was investigated using human fibroblast cells, and the results are presented in Figure 5d. On day 1, comparable cell viability was observed on the surface of GelMA and NCST hydrogel specimens. As shown in Figure 5d, somewhat improved cell viability was observed on the surface of NCST1, NCST2, and NCST3 hydrogels on day 2. However, comparable viability was perceived on the controller (tissue culture plate) and pristine GelMA specimen. On day 3, NCST1, NCST2, and NCST3 hydrogels demonstrated ample improvement in the cell viability (Figure 5d). It is attributed to the improved roughness, porosity, and uniform dispersion of f-CNOs within GelMA matrixes of NCST hydrogels. Cell viability measurements demonstrated that nanocomposite hydrogels with inimitable biocompatibility could be promising drug carriers. 

## 3. Materials and Methods

### 3.1. Materials 

Unless stated otherwise, all organic solvents were obtained from Sigma-Aldrich (St. Louis, MO, USA). Gelatin (Type A, 300 bloom from porcine skin), methacrylic anhydride (MA), 2-hydroxy-1-[4-(2-hydroxyethoxy) phenyl]-2-methyl-1-propanone (Irgacure 2959), citric acid, sodium phosphate monobasic dihydrate, trinitrobenzene sulfonic acid (TNBS), sodium bicarbonate, sodium dodecyl sulfate (SDS), γ-cyclodextrin, and doxorubicin (DOX) were purchased from Sigma-Aldrich (St. Louis, MO, USA). Human foreskin fibroblasts (ATCCVR CRL-2522) were procured from the American Type Culture Collection (ATCC, Manassas, VA, USA). Cell culture consumables and supplements were procured from Gibco Invitrogen (Carlsbad, CA, USA). CellTiter96^®^Aqueous One-Solution Cell Proliferation Assays was bought from, Promega, (Madison, WI, USA).

### 3.2. Synthesis of Key Building Blocks 

PAPMA-CNOs have synthesized using previous report (Scheme S1 in Supplementary Materials) [39]. ^39^ Citric acid cross-linked γ-cyclodextrin was prepared through previously reported method (Scheme S2 in Supplementary Materials) [40].

#### 3.2.1. Fabrication of GelMA Hydrogel

(a) Synthesis of GelMA: Methacrylated gelatin was prepared as described earlier (Figure 1a) [25,31]. Concisely, 10% (*w/v*) of gelatin solution was prepared in phosphate-buffered saline (PBS) at 50 °C. Then, MA was added until the target volume was reached at a rate of 0.5 mL/min to the gelatin solution under continuous stirring at 50 °C. The reaction mixture was allowed to react for 3 h. The degree of methacrylation on gelatin was determined by varying the concentration of MA. After that, the reaction was stopped by adding an excess of warm PBS (40 °C). The reaction mixture was dialyzed against distilled water using 12–14 kDa cutoff dialysis tubing for 5 days at 40 °C to remove unreacted materials. At this point, the obtaining solution was lyophilized and the attained GelMA was stored at 4 °C until further use. 

(b) Estimation of Degree of Methacrylation on Gelatin: The degree of methacrylation of lysine groups modified on the gelatin was determined by using the Habeeb method [45]. Briefly, 200 μg/mL (*w/v*) of freeze-dried GelMA was dissolved in 0.1 M sodium bicarbonate (pH 8.5). Next, 0.25 mL of TNBS solution (0.01% (*w/v*)) was added to 0.5 mL of each sample solution. The obtaining samples were nurtured at 37 °C for 3 h. Then, the reaction was stopped by adding 0.25 mL of 10% sodium dodecyl sulfate (SDS) and stabilized by adding 0.125 mL of 1 N hydrogen chloride (HCl). An optical density (OD) of specimens were measured using a U*V/v*is spectrophotometer at 335 nm. The degree of methacrylation was deliberated by comparing the amount of unreacted amino derivatives on gelatin and GelMA. 

(c) Fabrication of GelMA Hydrogel: Pristine GelMA hydrogel was fabricated as follows. Initially, freeze-dried GelMA (10% *w/v*) was dissolved in PBS containing 0.5% (*w/v*) of Irgacure 2959 as a photoinitiator. Then, the precursor solution was placed in poly(dimethylsiloxane) (PDMS) molds and subsequently exposed to UV irradiation (wavelength 360–480 nm) for different time intervals (1 to 10 min) to form the pristine GelMA hydrogel with different crosslinking densities. Then, the GelMA hydrogel was placed into PBS, and the temperature was decreased to 37 °C to occur physical gelation. To prepare the DOX-loaded GelMA hydrogel, 5 mg/mL of DOX was added to the above solution (pristine GelMA) before the photo-cross-linking step. DOX loaded hydrogels represented as GelMA/DOX. 

#### 3.2.2. Preparation of Inclusion Complex of DOX with Citric Acid Cross-Linked γ-Cyclodextrin (DOX@γ-CD IC) 

A mixture of citric acid cross-linked γ-cyclodextrin and DOX in a ratio of 1:1 (*w*/*w*) was dissolved and incubated for 24 h at room temperature in different pH solutions (pH 4.0 to 9.0). The resulting inclusion complex (DOX@γ-CD IC) solution was centrifuged at 25,000 rpm for 30 min to remove unbound free DOX. Thereafter, the DOX@γ-CD IC was frozen at −80 °C followed by lyophilized to attain solid DOX@γ-CD IC, which was used for further studies. The formation of DOX@γ-CD IC was confirmed by using FTIR, DSC, and TGA analyses. The quantity of DOX loaded into the inclusion complex was calculated by measuring the DOX in the freeze-dried solid and dissolving in pH 4.5 solution. Consequently, the obtained solution was analyzed by UV-vi spectroscopy. 

#### 3.2.3. Fabrication of GelMA/f-CNOs/DOX@γ-CD IC Hydrogels 

First, GelMA (10% *w/v*), DOX@γ-CD IC (5% *w/v*), and f-CNOs with different contents (1, 3, and 5 mg/mL) solutions were prepared in PBS separately. Then, GelMA (10% *w/v*), f-CNOs (1 mg/mL), and DOX@γ-CD IC (5% *w/v*) were transferred into PBS containing 0.5% (*w/v*) of irgacure 2959 as photoinitiator, and the mixture was ultra-sonicated for 5 min. The resulting precursor solution was then placed in PDMS molds and photo-cross-linked for different time intervals (1 to 10 min) to form GelMA/f-CNOs (1 mg/mL)/DOX@γ-CD IC (5% *w/v*) nanocomposite hydrogel with varying crosslinking densities. After that, the nanocomposite hydrogel was transferred into PBS, and the temperature was reduced to 37 °C to form physical gelation. The obtained composite hydrogels were rinsed with deionized (DI) water. The resulting composite hydrogels were air-dried then lyophilized. The dry composite hydrogels were stowed under inert conditions for further use. By using the same optimized fabrication conditions, GelMA/f-CNOs (3 mg/mL)/DOX@γ-CD IC (5% *w/v*), and GelMA/f-CNOs (5 mg/mL)/DOX@γ-CD IC (5% *w/v*) nanocomposite hydrogels were fabricated. Hereafter, GelMA/f-CNOs (1 mg/mL)/DOX@γ-CD IC (5% *w/v*), GelMA/f-CNOs (3 mg/mL)/DOX@γ-CD IC (5% *w/v*), and GelMA/f-CNOs (5 mg/mL)/DOX@γ-CD IC (5% *w/v*) nanocomposite hydrogels were denoted as NCST1, NCST2, and NCST3, respectively. NCST indicates nanocomposite hydrogels. 

### 3.3. Encapsulation Efficiency (EE) 

The encapsulation efficiency of the DOX in the hydrogels was calculated according to the following equation.

(1) $$\%\;\mathrm{EE}\;=\;\frac{Total\;amount\;of\;drug-Amount\;of\;unbound\;drug\;}{Total\;amount\;of\;drug}\times 100$$

The free and unbound DOX in the supernatant was calculated by UV-Vis spectroscopy. 

### 3.4. Characterizations of Composite Hydrogels

Scanning electron microscopy (SEM, ZEISS EVO^®^ MA 25, Ostalbkreis, Baden-Württemberg, Germany) was used to measure the surface morphological properties of hydrogel specimens. All the specimens were flash-frozen in liquid nitrogen followed by fractured. The fractured specimens were gold-coated prior to surface scanning. The functional groups of all the specimens were scrutinized using Fourier transform infrared spectroscopy (FTIR, PerkinElmer Universal ATR Sampling Accessory Frontier). A differential scanning calorimeter (DSC, TA Q2000, New Castle, DE) was used to measure the melting temperature and crystallinity of the samples at a scan rate of 10 °C/min with a temperature range of 25–300 °C under nitrogen atmosphere. A thermal gravimetric analysis (TGA, SDT Q600 (TA Instruments) was used to analyze the mass loss of the samples at a scan rate of 10 °C/min with a temperature range of 25–800 °C under nitrogen atmosphere. Hydrodynamic size, and Zeta potential measurements of specimens were studied by using Malvern Nano-ZS instrument, and Zetasizer software (version 7.12, Malvern Instruments Ltd., Worcestershire, WR14 1XZ, United Kingdom) was used to analyze the data. Dynamic light scattering (DLS) measurements were run in triplicate and averaged to acquire the final results. A tensile testing machine (Instron 3365, Instron, Norwood, MA) was utilized to measure the mechanical properties of samples. The dimensions of the sample were 33.0 mm × 6.0 mm × 2.0 mm, and the crosshead speed was 100 mm/min. All the experiments were run in triplicate. The surface wettability (water contact angles) of hydrogel specimens was measured using optical contact angle measurement (OCA 15EC). For this, 10 µL of deionized (DI) water droplet was slowly injected on the specimen surface by using a microdispenser. Triplicate experiments were carried out to attain the average results. Nitrogen (N_2_) adsorption/desorption isotherm measurements of specimens were determined by Quantachrome Autosorb AS-1 version-1.55 at 77 K. Before the analysis, the specimens were pre-treated under the high vacuum at 75 °C for overnight. The rotational and vibrational modes of the hydrogels were measured by using Raman spectroscopy (Renishaw inVia Raman microscope with a green laser of 514 nm). A rotational rheometer (RS600, Thermo Hakke, USA) was used to evaluate rheological measurements. All measurements made by using the specimens with dimensions of 20 mm × 1.0 mm (diameter × thickness). Storage modulus (G′) and loss modulus (G″) were recorded by using a frequency sweep mode at 20 C with the range of 10^−1^ to 10^1^ Hz in the viscoelastic region at 1% of strain.

#### 3.4.1. Equilibrium Swelling of Hydrogels 

To study equilibrium swelling of hydrogels, pre-weighed dry specimens with different crosslinking densities were submerged in a beaker containing Dulbecco’s Modified Eagle’s medium (DMEM) and allowed to soak for 24 h at 37 °C. At predetermined time intervals, the swollen hydrogels were taken out from the buffer, and removed excess water with filter paper. Immediately, the specimen was weighed (W_s_) with a microbalance. Then, samples were freeze-dried and weighed to determine the dry sample weight (W_d_). All the measurements were run in triplicate. The equilibrium-swelling ratio (ESR) was measured using the following equation [26,49]:

(2) $$\mathrm{SR}\;=\;\frac{W_{s}-W_{d}}{W_{d}}\times 100$$

where, *W_s_* is the weight of hydrogels at equilibrium state and *W_d_* is the weight of the hydrogels at the dry state.

#### 3.4.2. In Vitro Degradation of Hydrogel 

Degradation of hydrogels was studied with respect to weight loss. Initially weighed hydrogel specimens with different crosslinking densities (*W*_0_) were immersed in PBS and incubated for 25 days at 37 °C. Then, the samples were drawn out of the buffer solution at specified time intervals, washed with distilled water, then dehydrated in the desiccator for 12 h and weighed (*W_t_*) [26].

The weight loss ratio calculated as$\;100\times \frac{W0-Wt}{W0}$.

The weight remaining ratio was calculated as

(3) $$100-\left[100\times \frac{W0-Wt}{W0}\right]$$

#### 3.4.3. Porosity Measurements

The porosity of specimens with different crosslinking densities was measured from the SEM images of different hydrogels by using ImageJ software. For this, high-resolution TIFF images were used ad pores of relatively circular shapes were perceptible in the SEM images. At least 50 different measurements were documented for each hydrogel. Several dimensions were measured for each pore analysis and averaged together to calculate the final porosity of hydrogels.

#### 3.4.4. In vitro Drug Release from Hydrogels

UV-spectrophotometer (Agilent Technologies, 89090A, Santa Clara, CA, USA) was used to measure the in vitro DOX release from the hydrogels. Approximately, 30 mg of DOX-loaded hydrogel specimens were immersed in PBS (pH 4.5, 6.0, and 7.4) and gently incubated at 37 °C. At specified time intervals, 2 mL of DOX released buffer solution was withdrawn and replenished with 2 mL of fresh buffer to preserve the buffer volume constant. The drug release was measured. The release and concentration of DOX were determined by using UV spectrophotometer at 480 nm. The DOX release from specimens against release time was validated. Triplicate drug release experiments were carried. The following formula was used to calculate the drug release (%).

(4) $$\mathrm{Drug}\;\mathrm{release}\;(\%)\;=\;\frac{\mathrm{Weight}\;\mathrm{of}\;\mathrm{drug}\;\mathrm{released}\;}{\mathrm{Weight}\;\mathrm{of}\;\mathrm{drug}\;\mathrm{in}\;\mathrm{the}\;\mathrm{hydrogel}}\times 100$$

#### 3.4.5. Cytotoxicity Evaluation of Hydrogel Specimens 

Human fibroblast cells were utilized to assess the in vitro biocompatibility of hydrogel specimens. CellTiter96^®^AQueous One Solution Cell Proliferation Assay was used to measure cell viability. For this, disk-shaped (~6.3 mm in diameter) hydrogel specimens were cut into thin sections and sterilized using ethanol (70% *v/v*) followed by UV irradiation. The sterilized hydrogel specimens have decorated on 96 well plates. Next, human fibroblast cells (4.8 × 10^5^ cells/cm^2^) were seeded on the surface of sterilized hydrogel samples, which were cultivated in a DMEM medium with a 5% CO_2_ atmosphere at 37 °C. For this, DMEM medium was supplemented with 100 μg/mL penicillin, 100 μg/mL streptomycin, and 5% (*w/v*) fetal bovine serum. On the next day, the non-adherent cells were discarded and fibroblast cells cultured specimens were shifted into the fresh wells. Next, 20 µL of CellTiter was added over the specimens on day 1 to day 3 and cultivated for 60 min under dark conditions. A microplate reader (Synergy HT, BioTek, Winooski, VT, USA) was utilized to measure the absorbance of each well. The cell viability percentage of specimens was calculated from the absorbance of each well and controller (a tissue culture plate without hydrogel). The viability experiments were run in triplicate. 

### 3.5. Statistical Analysis

All the experiments were performed in triplicate, and the results were presented as mean ± standard deviation (SD). Tukey’s post hoc tests using Minitab 17 (Minitab, State College, PA, USA) and one-way analysis of variance (ANOVA) were executed to determine statistical analysis and significance was considered at *p* ≤ 0.05.

## 4. Conclusions

The current work presents the entrapment of citric acid crosslinked γ-cyclodextrin (γ-CD) contained DOX within GelMA/f-CNOs hydrogels by using a photo-crosslinking method. DOX formed an inclusion complex with γ-CD and was then encapsulated into GelMA/f-CNOs with enhanced solubility and controlled release of DOX. The obtained composite hydrogels exhibited improved thermal, wettability, swelling, and mechanical properties. The crosslinking density, high content of f-CNOs, multiple interfaces, and stress transfer between GelMA and f-CNOs are the major factors that amended the mechanical properties of composite hydrogels. The composite hydrogels were extensively sensitive towards pH, prolonged, and the highest drug release was observed from the NCST3 system over 18 days at pH 4.5. Besides this, composite hydrogels presented good cell viability with human fibroblast cells. Overall, the results reveal the γ-cyclodextrin integrated GelMA/f-CNOs based pH-triggered anticancer drug delivery platform; which may be advantageous in the future to deliver the encapsulated therapeutic drug molecules in a controlled manner.

## Data Availability

Data is contained within the article or supplementary material.

## Supplementary Materials

The following are available online at https://www.mdpi.com/article/10.3390/ph14040291/s1, Figure S1: (a) Hydrodynamic size distribution of f-CNOs determined by using DLS at room temperature. (b) N_2_ gas adsorption/desorption isotherms, and (c) pore size dissemination of f-CNOs, respectively. Data are connoted as mean ± SD, (*n* = 3); Figure S2: (a) DOX loading efficiency of γ-CD at pH 4.5 to 9.0, and (b) percentage of DOX retained on γ-CD at pH 4.5, 7.4, and 9.0. Data implied as mean ± SD, *n* = 3; Figure S3: Absorption spectra of DOX (1.6 × 10^−4^ M) in PBS in the range of pH 4.5–9.5; Figure S4: (a) FTIR, (b) DSC, (c) TGA, (d) fluorescence titration of DOX (1.0 × 10^−5^ mol/L) with various concentration of γ-CD, and (e) Benesi-Hildebrand plot of 1/F - F0 versus 1/[γ-CD] at room temperature, λex = 480 nm.; Figure S5: Degradation, swelling, mechanical, and porosity of hydrogels under different crosslinking time intervals: (a) Degradation, (b) swelling, (c) pore diameter, and (d) tensile stress of neat GelMA, NCST1, NCST2, and NCST3 hydrogels under 100, 250, and 500 sec UV exposure time, respectively; Figure S6: Degrees of crosslinking (%) of pristine GelMA, and NCST hydrogels after crosslinking under UV light; Figure S7: Raman spectra of Neat f-CNOs, and NCST hydrogels before and after degradation test for 48 h; Scheme S1: Synthesis of poly (N-(4-aminophenyl)methacrylamide)) (PAPMA-CNOs = f-CNOs) from (N-(4-aminophenyl)methacrylamide)) and COOH-CNOs; Scheme S2: Synthesis of citric acid cross-linked γ-cyclodextrin from citric acid monohydrate and γ-cyclodextrin in the presence of sodium dihydrogen phosphate.
