# Supplementary material for: Covalently Functionalized Carbon Nano-Onions Integrated Gelatin Methacryloyl Nanocomposite Hydrogel Containing γ-Cyclodextrin as Drug Carrier for High-Performance pH-Triggered Drug Release
Source: Pharmaceuticals (Basel). 2021 Mar 25;14(4):291. doi: 10.3390/ph14040291 (PMC8064464; doi:10.3390/ph14040291)
Supplement: Supplementary file 1 [file pharmaceuticals-14-00291-s001.pdf]

Supporting Information

Covalently Functionalized Carbon Nano-onions Integrated Gelatin Methacryloyl Nanocomposite Hydrogel Containing γ-Cyclodextrin as Drug Carrier for High Performance pH-Triggered Drug Release

Narsimha Mamidi[a,*], Ramiro Manuel Velasco Delgadillo[a], Enrique V. Barrera[b]

[a]Department of Chemistry and Nanotechnology, School of Engineering and Science, Tecnologico de Monterrey, Monterrey, Nuevo Leon-64849, Mexico

[b]Department of Materials Science and Nanoengineering, Rice University, Houston, TX 77005, USA

Email: nmamidi@tec.mx; Phone: + 81-83582000-4593

1. Synthesis of poly (N-(4-aminophenyl)methacrylamide)) (PAPMA-CNOs = f-CNOs)

The synthesis of f-CNOs was achieved by using previous report.[1] The procedure was slightly modified.

(a) Synthesis of N-(4-aminophenyl)methacrylamide (APMA): *p*-Phenylenediamine (1.0 g, 9.25 mmol) and triethylamine (1.3 mL, 9.25 mmol) were dissolved in anhydrous DCM, and the reaction mixture was probe sonicated for 50 min. Subsequently, 903 μL (9.25 mmol) of methacryloyl chloride was added dropwise into the above reaction mixture, and the probe sonicated for an additional 60 min. Then, the reaction mixture was washed twice with NaOH solution followed by water to neutralize the pH. The resulting crude product was purified by silica gel column chromatography with a gradient solvent system of ethyl acetate/hexane (2:1) and 2.0 vol.% of trimethylamine. The obtained APMA was characterized by using NMR spectroscopy. ^{1}H NMR (500 MHz, DMSO-d_6) δ_{ppm}: 9.33 (br, 1H), 7.29 (d, 2H), 6.61 (d, 2H), 5.80 (s, 1H), 5.43 (s, 1H), 4.86 (s, 2H), 2.01 (s, 3H); ^{13}C NMR (125 MHz, DMSO-d_6) d (ppm): 166.92, 144.60, 141.61, 128.25, 124.21, 119.91, 114.80, 18.03.

(b) Synthesis of poly (N-(4-aminophenyl)methacrylamide)) (PAPMA): 1.0 g of APMA (5.68 mmol), 1.0 wt.% of AIBN, and 15 mL of anhydrous THF were transferred into a Schlenk tube (50 mL). Four freeze-pump-thaw cycles were made, and the reaction mixture was stirred at 65 °C for 60 min. Then, around 100 mL of diethyl ether was added into the above reaction mixture to precipitate the polymer. The resulting precipitate was filtered and carefully washed with excess

dichloromethane (DCM) to yield PAPMA. Finally, PAPMA was analyzed by NMR. ^{1}H-NMR (500 MHz, DMSO-d_6): δ_{ppm}: 7.43 (2H, br s, *o,o'*-ArH), 6.33 (2H, br s, *m,m'*-ArH), 2.05 (2H, br peak, CH_2 polymeric), 1.10-0.93 (3H, br peak, CH_3 aliphatic). ^{13}C-NMR (125 MHz, DMSO-d_6): δ_{ppm}: 164.30, 153.41, 131.81, 124.50, 123.31, 121.93, 119.41, 116.85, 45.38, 18.70.

(c) Synthesis of PAPMA-CNOs (f-CNOs):

Initially, COOH-CNOs (50 mg), EDC (285 mg), and NHS (175 mg) were dissolved in 50 mL of anhydrous DMF, and probe sonication was continued for 60 min to incite the carboxyl groups of CNOs. Then, 100 mg of PAPMA in anhydrous DMF was added into the above mixture, and probe sonication was continued for a further 120 min. After that, the reaction was stopped and centrifuged at 15,000 rpm to obtain PAPMA-CNOs (f-CNOs) as black cake. The f-CNOs were thorough with DMF/trimethylamine (9.9:0.1) and stored in a refrigerator at 4 °C. The f-CNOs were analyzed by NMR. ^{1}H-NMR (500 MHz, DMSO-d_6): δ_{ppm}: 7.10 (2H, br s, *o,o'*-ArH), 6.42 (2H, br s, *m,m'*-ArH), 2.01-1.21 (2H, br peak, CH_2 polymeric), 1.39-0.90 (3H, br peak, CH_3 polymeric). ^{13}C-NMR (125 MHz, DMSO-d_6): δ_{ppm} 174.50, 167.41, 153.73, 131.82, 124.10, 123.21, 122.30, 119.02, 117.30, 44. 10, 18.92.

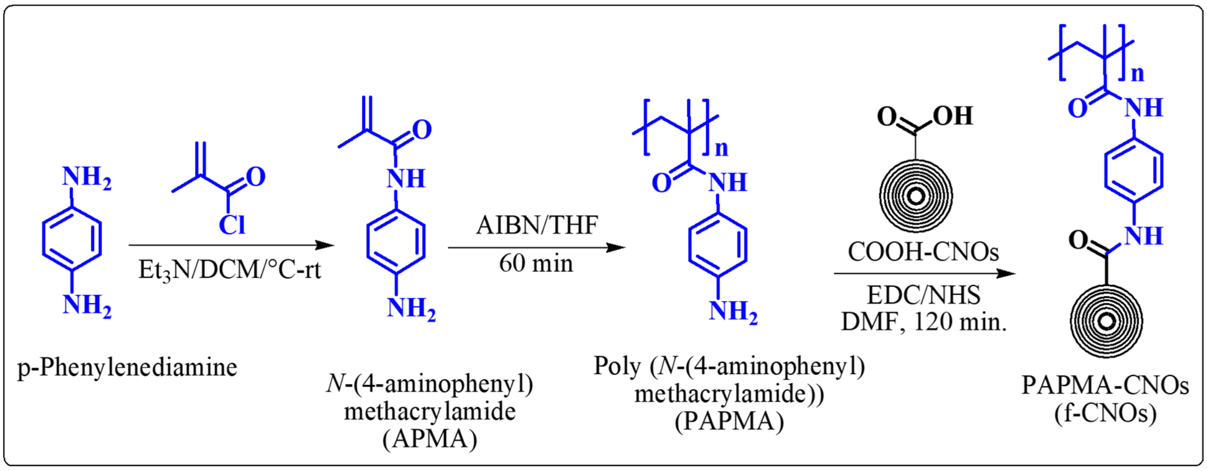

Scheme S1. Synthesis of poly (N-(4-aminophenyl)methacrylamide)) (PAPMA-CNOs = f-CNOs) from (N-(4-aminophenyl)methacrylamide)) and COOH-CNOs.

2. Size, Surface Area and Pore Distribution Measurements of f-CNOs

The physicochemical properties of f-CNOs are essential characteristics for hybrid hydrogel material. Therefore, hydrodynamic size, N_2 sorption, and pore distribution of f-CNOs were measured, and the results were presented in Figure S1. As revealed by DLS analysis, f-CNOs

exhibited the hydrodynamic size of 87 ± 4 nm (Figure S1a). As shown in Figure S1b, f-CNOs showed a type IV adsorption isotherm with an apparent hysteresis loop, illuminating a diversity of pores and the presence of mesopores and micropores.[2] The surface area within the nanoparticles was found to be 358.3 $m^2.g^{-1}$ for f-CNOs. The pore-size distribution of f-CNOs illustrated in Figure 2c designates that the pore size is mostly between 1.81 and 4.11 nm.

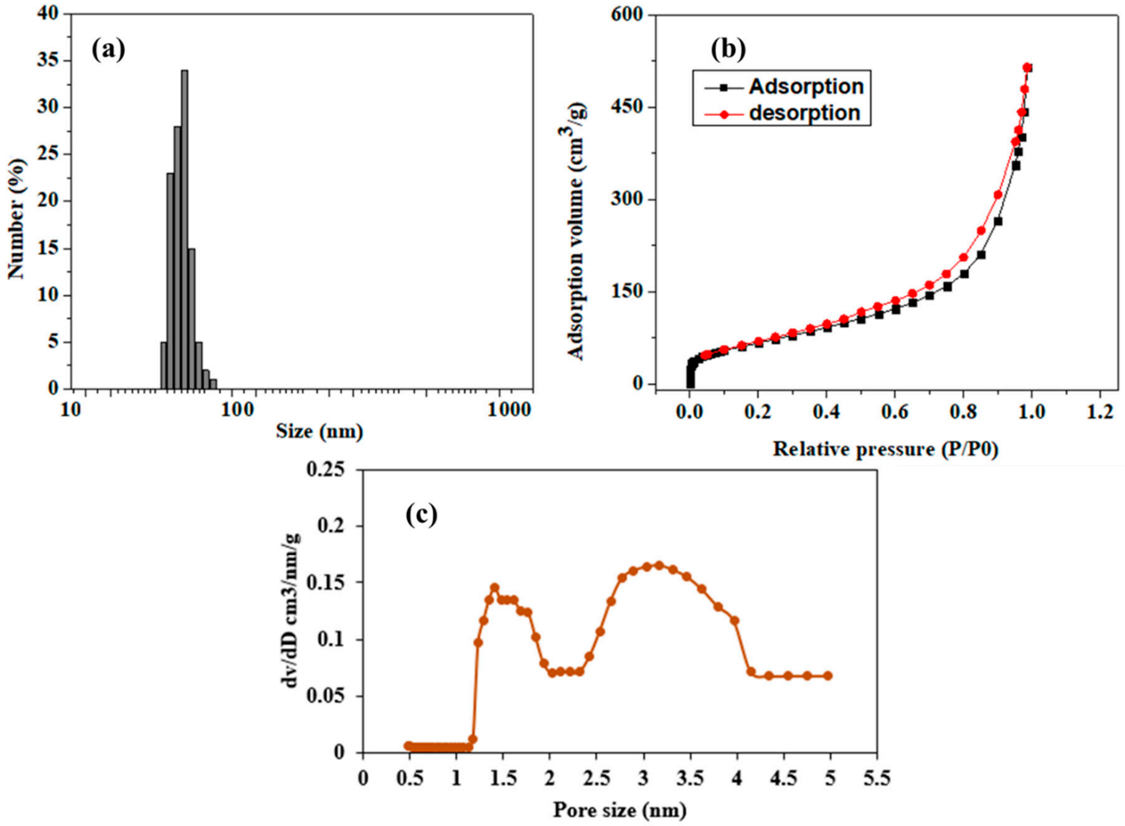

Figure S1. (a) Hydrodynamic size distribution of f-CNOs determined by using DLS at room temperature. (b) N_2 gas adsorption/desorption isotherms, and (c) pore size dissemination of f-CNOs, respectively. Data are connoted as mean ± SD, (n = 3).

3. Synthesis of critric acid cross-linked γ-cyclodextrin

The synthesis of critric acid cross-linked γ-cyclodextrin was synthesized using the previous protocol.[3] "Briefly, 1 g of γ-cyclodextrin (γ-CD), 3 g of citric acid monohydrate, 0.3 g of sodium dihydrogen phosphate, and 70 mL of deionized (DI) water were mixed in a beaker and stirred in a hot water bath to attain homogeneity. Then, the reaction mixture was poured into a Petri dish followed by heating in a drying oven at 140 °C for 30 min to form yellowish gel material. After

cooling to room temperature, the crude product was purified by soaking in 300 mL of water (5-6 times). Then, suction filtration was performed followed by dried at 60 °C for overnight. Then, the cross-linked γ-CD was characterized using FTIR spectroscopy".

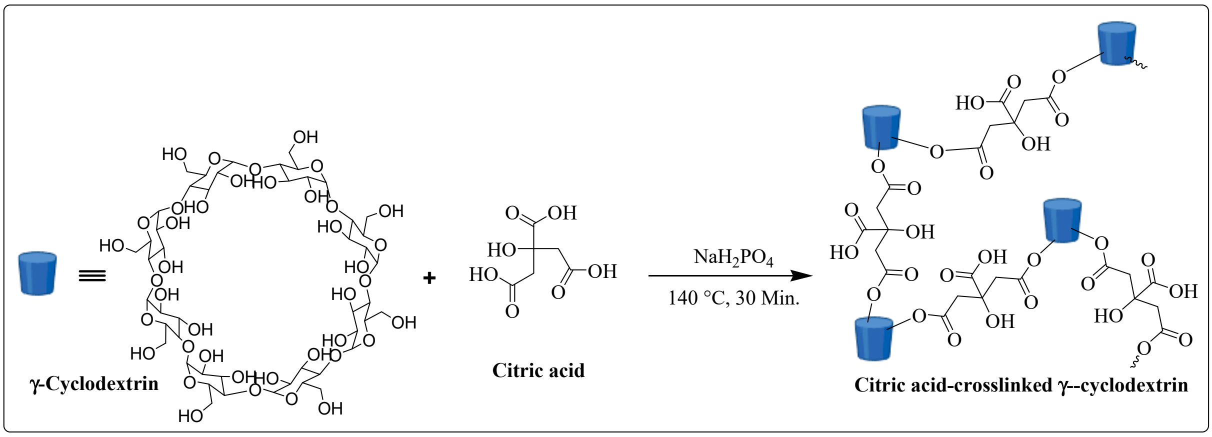

Scheme S2. Synthesis of citric acid cross-linked γ-cyclodextrin from citric acid monohydrate and γ-cyclodextrin in the presence of sodium dihydrogen phosphate.

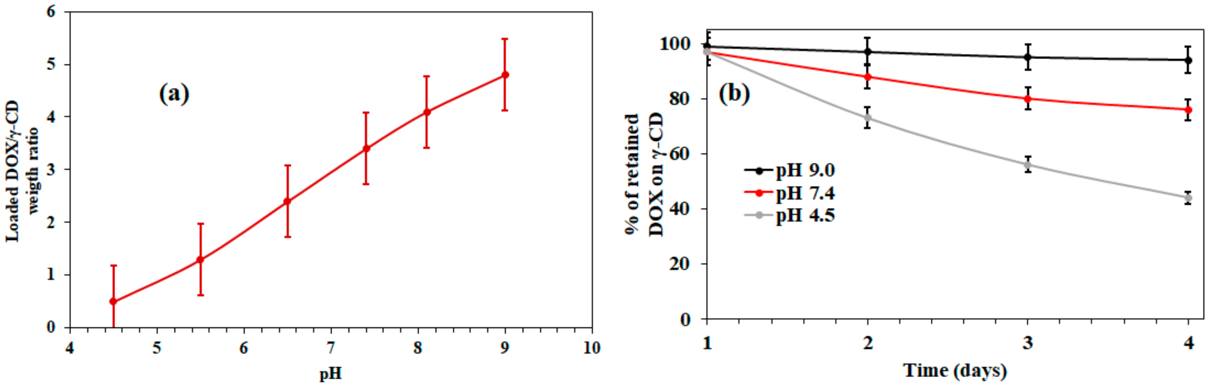

Figure S2. (a) DOX loading efficiency of γ-CD at pH 4.5 to 9.0, and (b) percentage of DOX retained on γ-CD at pH 4.5, 7.4, and 9.0. Data implied as mean ± SD, n = 3.

4. Stability DOX in acidic and basic pH solutions

"We measured drug release under pH 4.5, pH 6.0, and pH 7.4. Therefore, we have recorded UV-Vis spectra of DOX in different pH solutions (4.5, 6.0, 7.4, 8.0, 9.0, and 9.5) and we did not find any degradation of DOX. The absorption band at 485 nm shows the presence of DOX and its stability in different pH solutions (Figure S3). The molar concentration (1.6 x 10-4 M) of DOX

was kept constant in PBS (pH 4.5 – 9.5). Similar results were also absorbed in the previous research reports".[4-6]

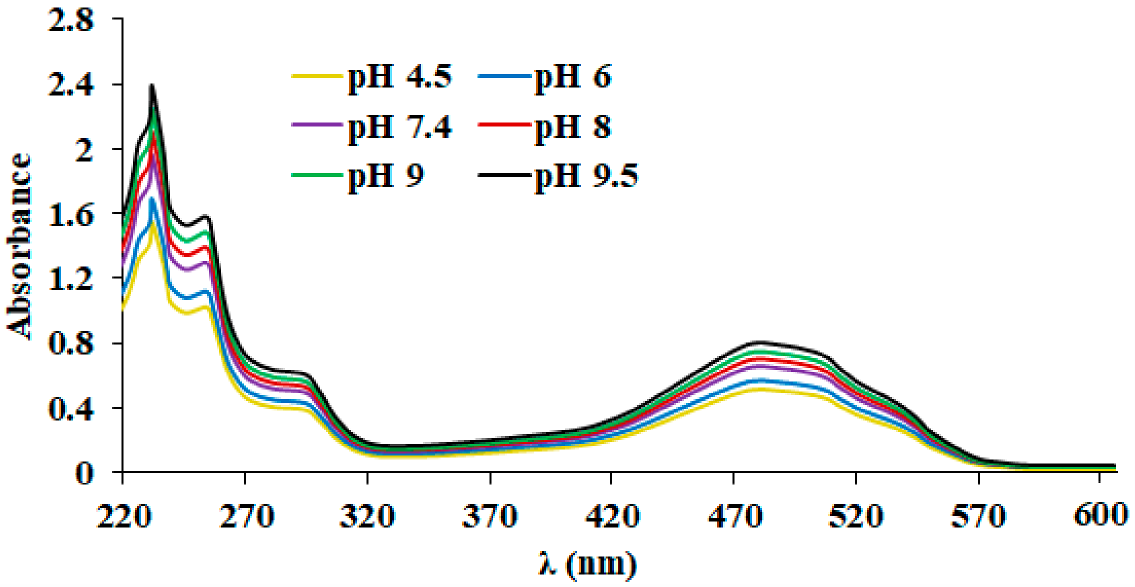

Figure S3. Absorption spectra of DOX (1.6 x 10^{-4} M) in PBS in the range of pH 4.5 – 9.5.

5. Confirmation Studies of Inclusion Complex (DOX@γ-CD IC)

"It is known that the drug/cyclodextrin inclusion complexes (Drug@CD ICs) can be confirmed by FTIR, NMR, XRD, DSC, TGA, UV-Vis, Fluorescence, Association constants determination by Benesi-Hildebrand method and molecular modeling (Gaussian model).[7-11] In the part of current study, we have used FTIR, DSC, TGA, fluorescence and Benesi-Hildebrand equation to confirm the formation of DOX@γ-CD IC. The results were presented in Figure S4. Initially, the formation of DOX@γ-CD IC was confirmed by using FTIR measurements. The major characteristic peaks of citric acid cross-linked γ-CD were observed at 3271, 2926, 1735, 1284, and 1018 cm^{-1} corresponding to the O-H stretching, C-H stretching, C=O stretching of ester groups, C-O-C glycosidic bridge, and C-O stretching vibrations of γ-cyclodextrin, respectively (Figure S4a). The FTIR spectrum of neat DOX showed characteristic bands at 1680, 1568, 1440, 1340, 1252 and 1050 cm^{-1} corresponding to quinone, anthracyclines, and C-O stretching vibrations, respectively. Bsesides, two broad peaks were observed at 3268, and 2929 cm-1 for $-NH_2$/O-H stretching, and C-H stretching of DOX, respectively. The FTIR spectrum of DOX@γ-CD IC was illustrated in Figure 1a, and DOX@γ-CD IC exhibited the overlapping of FTIR peaks of γ-CD and DOX. However, the absorption peaks of DOX at 1653, 1530, 1287 observed for DOX@γ-CD IC, which

revealed the presence of DOX in the inclusion complex. Furthermore, characteristic absorption peaks of DOX and γ-CD were somewhat shifted for DOX@γ-CD IC when compared to neat DOX and γ-CD, which suggesting the host-guest interactions between DOX and γ-CD in the inclusion complex. Similar results were observed for most of the CD IC systems.[12]

The DOX@γ-CD IC was further characterized by DSC to verify whether the DOX was incorporated inside the γ-CD cavity or not (Figure S4b). It is known that the melting point (T_m) and decomposition (T_d) temperatures of CD molecules would be observed if there were any un-complexed CD molecules present in the CD ICs.[13] Thus, DSC analysis was performed for neat DOX, γ-CD, and DOX@γ-CD IC, and all curves were illustrated in Figure S3b. Neat DOX showed three major peaks at 205, 217, and 237 °C corresponding to the T_m of different crystalline structures and T_d of DOX.[14] Whereas the thermogram of DOX@γ-CD IC did not display any melting for neat DOX, which demonstrates that the DOX molecules were fully complexed with the γ-CD. Thus, the absence of DOX in the DSC thermogram of DOX@γ-CD IC comprises noteworthy evidence for the complete inclusion complexation of DOX with γ-CD.

Besides, TGA analysis was performed for DOX@γ-CD IC to explore the thermal stability as well as evaporation of DOX incorporated inside the γ-CD cavity (Figure S4c). It is worth mentioning that the thermal stability, as well as the evaporation of the volatile guest molecules, change to high temperature upon inclusion complexation due to host-guest interactions.[15] The TGA thermogram of neat DOX showed a major weight loss in the range of 175–235 °C, indicating that DOX has a slight volatile behavior. DOX@γ-CD IC exhibited three weight losses, the first weight loss around 105 °C corresponds to the water loss, and the second weight loss between 195 to 300 °C is due to the evaporation of DOX. The main weight loss above 300 °C corresponds to the thermal decomposition of γ-CD.[16] Thus, the TGA curve of DOX@γ-CD IC displayed that the thermal degradation of DOX occurred over a higher temperature range (195 to 300 °C), conforming to the thermal stability of DOX was augmented due to the inclusion complexation with γ-CD. Overall, the TGA results demonstrated that the thermal stability of DOX was shifted to a much higher temperature because of the strong interactions between DOX and the γ-CD cavity in the DOX@γ-CD IC.

As shown in Figure S4d, fluorescence titration of DOX (1.0×10^{-5}) was conducted using different concentrations of γ-CD ranging from 5.0×10^{-4} to 9.0×10^{-3}. As the γ-CD concentration was

increased, the fluorescence intensity of DOX was gradually increased (Figure S4d). The apparent formation constant (K_f) of DOX@γ-CD IC was determined by exploring fluorescence intensity at 555 nm in terms of the modified Benesi-Hildebrand equation for 1:1 stoichiometry:[10,11]

$$\frac{1}{F - F_0} = \frac{1}{(F_\infty - F_0)K_f[\gamma - CD]} + \frac{1}{F_\infty - F_0}$$

Where F is the observed fluorescence intensity of the DOX at each γ-CD concentration performed. F_0 and F_∞ indicates the fluorescence intensity in the absence and presence of γ-CD, respectively. At a concentration of (1.0×10^{-5}) of DOX, using a plot of 1/F - F_0 versus 1/[γ-CD], the linear relationship that describes the stoichiometric coefficient, and subsequently, K_f value is obtained. When values of 1/(F - F_0) were plotted as a function of 1/[γ-CD], a nonlinear curve was perceived that revealed a complex stoichiometry ratio of 1:1 (Figure S4e).[10,11] The analysis of the plot provides the K_f value of 3.35×10^4 $mol^{-1}L$. Based on these results, it is hypothesized that the DOX molecules are assembled within a cavity of γ-CD through van hydrophobic interaction, der Waals forces, and hydrogen bonding interactions between DOX and γ-CD, which confirm the formation DOX@γ-CD IC".

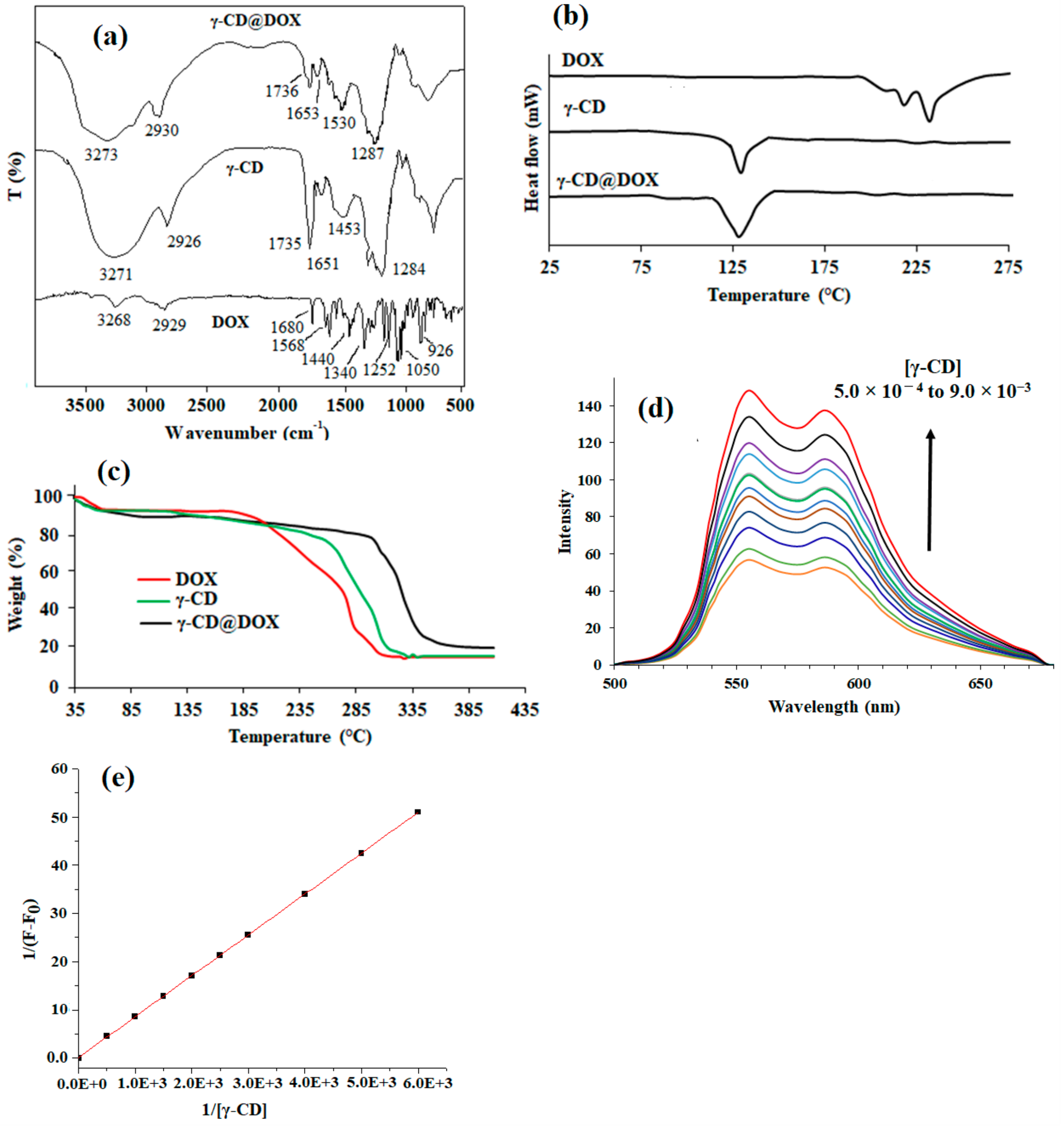

Figure S4. (a) FTIR, (b) DSC, (c) TGA, (d) fluorescence titration of DOX (1.0 x 10^{-5} mol/L) with various concentration of γ-CD, and (e) Benesi-Hildebrand plot of 1/F - F_0 versus 1/[γ-CD] at room temperature, λ_{ex}=480 nm.

6. The Influence of Crosslinking Density on Properties of Hydrogels

It is known that the degree of crosslinking density significantly influences the physicochemical characteristics of GelMA based hydrogels.[17] For this, degradation, swelling, porosity, and mechanical properties of GelMA and NCST hydrogels were studied with a function of UV exposure times (100, 250, and 500 sec). The results were presented in Figure S5. As seen in Figure S5a, the degradation rate of GelMA was gradually decreased, and it was inversely proportional to the crosslinking density and the concentration of f-CNOs. To confirm the crosslinking density of neat GelMA and NCST hydrogels, the swelling ratio was evaluated, which could be directly reflected the crosslinking density of the hydrogels.

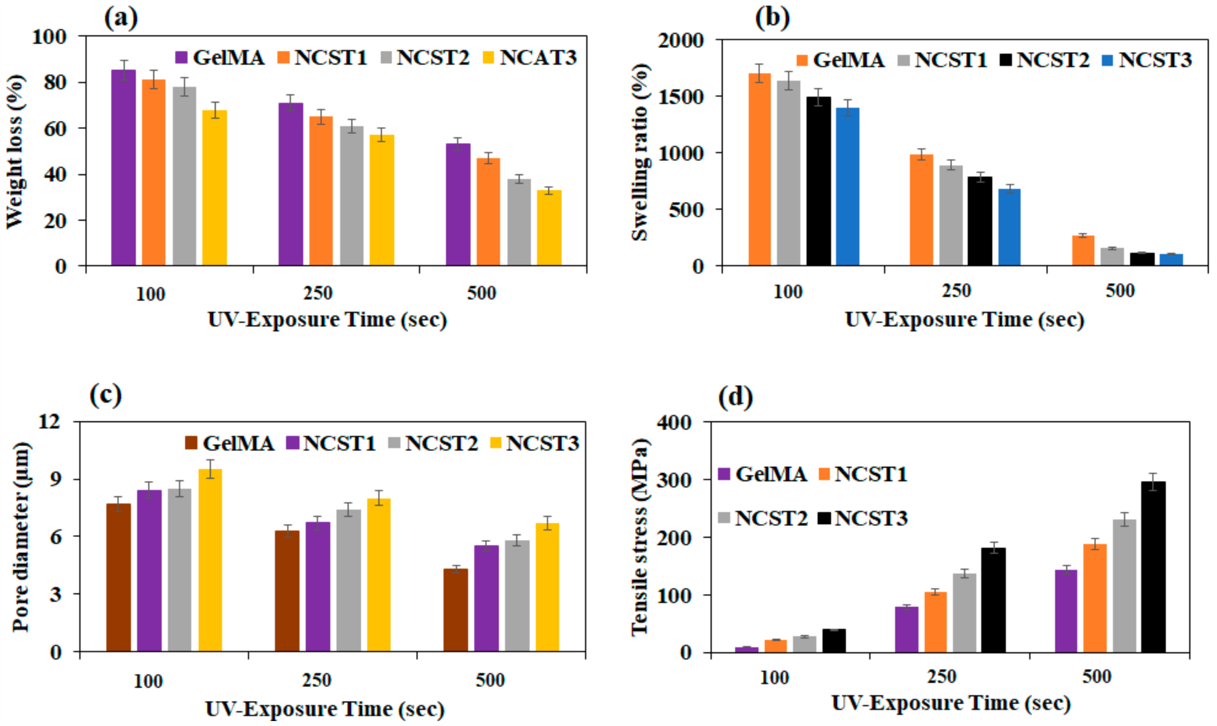

Figure S5. Degradation, swelling, mechanical, and porosity of hydrogels under different crosslinking time intervals: (a) Degradation, (b) swelling, (c) pore diameter, and (d) tensile stress of neat GelMA, NCST1, NCST2, and NCST3 hydrogels under 100, 250, and 500 sec UV exposure time, respectively.

As showed in Figure S5b, pristine GelMA hydrogels showed a higher swelling ratio compare to the NCST hydrogels when exposed to UV light for 100, and 250 sec, respectively. This could be due to the high number of MA groups of GelMA are cross-linked under UV light, whereas with

the incorporation of f-CNOs into GelMA, the MA groups of GelMA might have involved in electrostatic interactions with f-CNOs networks, which shielded MA groups of GelMA from UV light. Therefore, the swelling ratio of NCST hydrogels somewhat lower than that of GelMA hydrogel. However, under longer time (500 sec) UV exposure, all the hydrogels displayed comparable swelling profiles. As showed in Figure S5c, the porosity of hydrogels was greatly influenced by the crosslinking density as well as the content of f-CNOs. Besides, the tensile stress of GelMA and NCST hydrogels was improved by increasing the crosslinking density and f-CNOs concentrations (Figure S5d).

7. Determination of Crosslinking Degree

The calculation of the crosslinking degree was performed by modifying TNBS (2,4,6-trinitrobenzene sulphonic acid) method.[18,19] The perception of this method is to enumerate the non-crosslinked amide ends of gelatin through their chemical reactions with TNBS. Briefly, 4 mg of crosslinked hydrogel scaffold was taken into a test tube. Then, 1 mL of 0.5% w/v TNBS solution, and 1 mL of 4% w/v $NaHCO_3$ solution (pH 8.5) were added. The resulting solution was heated at 40 °C for 120 min followed by 2 mL of 6N HCl was added, and the temperature was raised to 60 °C for 90 min. The obtaining solution was diluted with 4 mL of deionized water, and the absorbance of the solution was measured by using UV spectroscopy. The degree of crosslinking of scaffolds was calculated by using the following equation, and the percentage values were presented as mean ± standard deviation (n = 3).

$$\text{Degree of crosslinking (\%)} = \left(1 - \frac{A_c}{A_n}\right) \times 100$$

Where A_c denotes the absorbance of the crosslinked and A_n represent non-crosslinked samples, respectively.

Initially, GelMA and NCST scaffolds were cross-linked by UV irradiation by varying the exposure time from 100 to 600 sec. The degrees of crosslinking of hydrogel scaffolds crosslinked by UV irradiation were illustrated in Figure S6. The degree of crosslinking of neat GelMA was gradually increased along with UV exposure times. Specifically, pristine GelMA hydrogel showed a maximum of 76 and a minimum of 15% of the degree of crosslinking in 600 and 100 sec, respectively (Figure S6). Whereas 72, 70, and 69% of degrees of crosslinking were found from the

NCST1, NCST2, and NCST3 hydrogels over 600 sec, respectively (Figure S6). These results demonstrated that the NCST hydrogels exhibited somewhat lower degrees of crosslinking compared with neat GelMA hydrogel. This could be due to the high number of cross-linking sites (MA groups) present on the surface of GelMA were exposed to the UV light, whereas cross-linking sites present in NCST hydrogels might have not been completely exposed to UV light.

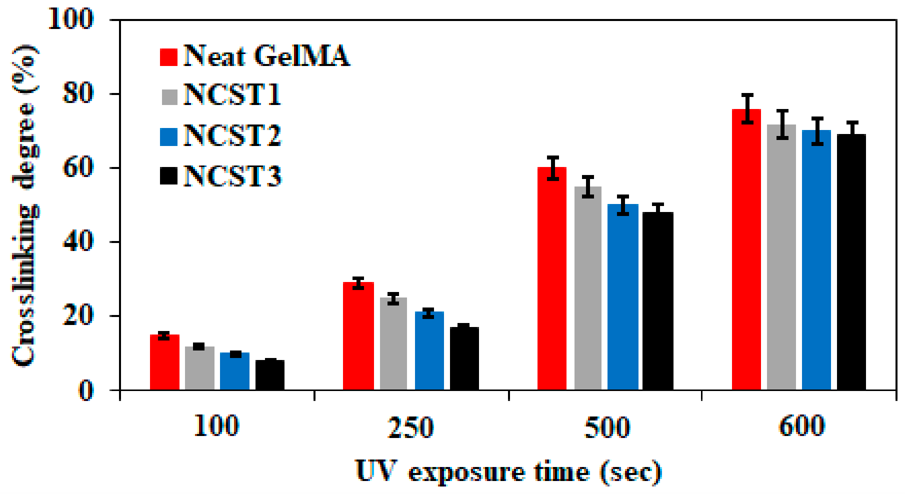

Figure S6. Degrees of crosslinking (%) of pristine GelMA, and NCST hydrogels after crosslinking under UV light.

8. Raman Spectroscopy

To validate that NCST hydrogels are useful hybrid composites for drug delivery and cellular measurements, it is essential to endorse that augmenting the mechanical properties by f-CNOs inclusion does not affect the favorable features of pristine GelMA. Raman spectroscopy was employed to compare the main characteristic graphite peaks of neat f-CNOs and NCST hydrogels before and after degradation (Figure S7). Raman spectra of NSCT1 and CNCST3 hydrogels before and after degradation have exhibited similar D and G bands. Particularly, the D band corresponding to the disorder in carbon systems was observed at 1357 cm^{-1}, whereas the G band for vibration modes of SP^2 bonded carbon atoms was observed at 1581 cm^{-1}. These results of CNST1 and CNST3 hydrogels demonstrating the structural integrity of f-CNOs seemed unaffected after the integration with GelMA and CD or by the degradation measurements. Similar results were observed from the GelMA-GO and GelMA-CNTs hydrogels under collagenase degradation.[20,21]

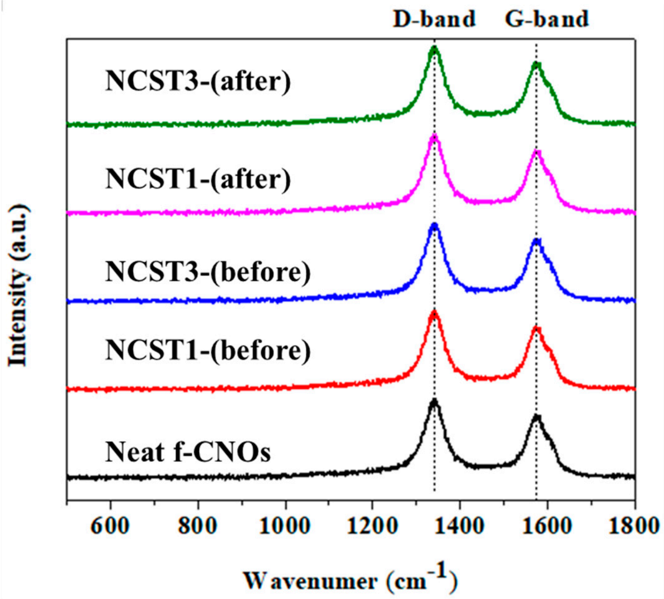

Figure S7. Raman spectra of Neat f-CNOs, and NCST hydrogels before and after degradation test for 48 h.

References

1. Mamidi, N.; Delgadillo, R. M. V.; Ortiz, A. G.; Barrera, E. V. Carbon Nano-Onions Reinforced Multilayered Thin Film System for Stimuli-Responsive Drug Release. *Pharm. 2020, Vol. 12, Page 1208* **2020**, *12* (12), 1208. https://doi.org/10.3390/PHARMACEUTICS12121208.
2. Hassan, A.F. Synthesis of carbon nano-onion embedded metal–organic frameworks as an efficient adsorbent for cadmium ions: kinetic and thermodynamic studies. *Environ Sci Pollut Res*, 2019, *26*, 24099–24111. https://doi.org/10.1007/s11356-019-05581-5
3. Anand, R.; Malanga, M.; Manet, I.; Manoli, F.; Tuza, K.; Aykaç, A.; Ladavière, C.; Fenyvesi, E.; Vargas-Berenguel, A.; Gref, R.; Monti, S. Citric Acid-γ-Cyclodextrin Crosslinked Oligomers as Carriers for Doxorubicin Delivery. *Photochem. Photobiol. Sci.* **2013**, *12* (10), 1841–1854. https://doi.org/10.1039/c3pp50169h.
4. Arora, H. C.; Jensen, M. P.; Yuan, Y.; Wu, A.; Vogt, S.; Paunesku, T.; Woloschak, G. E. Nanocarriers Enhance Doxorubicin Uptake in Drug-Resistant Ovarian Cancer Cells. *Cancer Res.* **2012**, *72*, 769–778.

5. Wang, H., Liang, Y., Yin, Y. et al. Carbon nano-onion-mediated dual targeting of P-selectin and P-glycoprotein to overcome cancer drug resistance. *Nat Commun* **2021**, *12*, 312. https://doi.org/10.1038/s41467-020-20588-0
6. Anand R.; Ottani S.; Manoli F.; Manet I.; Monti S. A closeup on doxorubicin binding to c-cyclodextrin: An elucidating spectroscopic, photophysical and conformational study. *RSC Adv.* **2012**; *2*: 2346-2357
7. Karpkird, T.; Manaprasertsak, A.; Penkitti, A.; Sinthuvanich, C.; Singchuwong, T.; Leepasert, T. A novel chitosan-citric acid crosslinked beta cyclodextrin nanocarriers for insoluble drug delivery, *Carbohydrate Research, **2020**, 498,108184,* https://doi.org/10.1016/j.carres.2020.108184.
8. Kost, B.; Brzeziński, M.; Zimnicka, M.; Socka, M.; Wielgus, E.; Słowianek, M.; Biela, T. PLA Stereocomplexed Microspheres Modified with Methyl-β-Cyclodextrin as an Atropine Delivery System. Synthesis and Characterization. *Materials Today Communications*, **2020**, *25*, 101605.
9. Neacșu, A. Physicochemical investigation of the complexation between γ-cyclodextrin and doxorubicin in solution and in solid state, *Thermochimica Acta,* **2018**, 661, 51-58.
10. Mesplet, N.; Morin, P.; Ribet, J-P. Spectrofluorimetric study of eflucimibe-γ-cyclodextrin inclusion complex, *European Journal of Pharmaceutics and Biopharmaceutics*, **2005**, 59, 523-526.
11. Peng, M.; Liu, Y.; Zhang, H.; Cui, Y.; Zhai, G.; Chen, C. Photostability study of doxorubicin aqueous solution enhanced by inclusion interaction between doxorubicin and hydroxypropyl-β-cyclodextrin," *Chinese Journal of Chemistry*, **2010**, *28*, 1291-1295.
12. Singh, R.; Bharti, N.; Madan, J.; Hiremath, S. Characterization of cyclodextrin inclusion complexes-a review. *J. Pharm. Sci. Technol.* **2010**, *2*, 171–183.
13. Giordano, F.; Novak, C.; Moyano, J. R. Thermal analysis of cyclodextrins and their inclusion compounds. *Thermochim. Acta* **2001**, *380*, 123–151
14. Missirlis, D.; Kawamurab, R.; Tirelli, N.; Hubbell, J. A. Doxorubicin encapsulation and diffusional release from stable, polymeric, hydrogel nanoparticles. *Eur. J. Pharm. Sci.* **2006**, *29*, 120. https://doi.org/10.1016/j.ejps.2006.06.003

15. Marcolino, V. A.; Zanin, G. M.; Durrant, L. R.; Benassi, M. D. T.; Matioli, G. Interaction of curcumin and bixin with β-cyclodextrin: complexation methods, stability, and applications in food. *J. Agric. Food Chem*. **2011**, *59*, 3348–3357.
16. Kayaci, F.; Uyar. T. Solid Inclusion Complexes of Vanillin with Cyclodextrins: Their Formation, Characterization, and High-Temperature Stability. *J. Agric. Food Chem*. **2011**, *59*, 21, 11772–11778, dx.doi.org/10.1021/jf202915c
17. Yue, K.; Trujillo-de Santiago, G.; Alvarez, M. M.; Tamayol, A.; Annabi, N.; Khademhosseini, A. Synthesis, Properties, and Biomedical Applications of Gelatin Methacryloyl (GelMA) Hydrogels. *Biomaterials* **2015**, *73*, 254–271. https://doi.org/10.1016/j.biomaterials.2015.08.045.
18. Choi, Y. S.; Hong, S. R.; Lee, Y. M.; Song, K. W.; Park, M. H.; Nam, Y. S. Study on Gelatin-Containing Artificial Skin: I. Preparation and Characteristics of Novel Gelatin-Alginate Sponge. *Biomaterials* **1999**, *20*, 409−417
19. Nagai, N.; Yunoki, S.; Suzuki, T.; Sakata, M.; Tajima, K.; Munekata, M. Application of cross-linked salmon atelocollagen to the scaffold of human periodontal ligament cells. *J. Biosci. Bioeng.* **2004**, *97*, 389−394.
20. Shin, S. R.; Aghaei-Ghareh-Bolagh, B.; Dang, T. T.; Topkaya, S. N.; Gao, X.; Yang, S. Y.; Jung, S. M.; Oh, J. H.; Dokmeci, M. R.; Tang, X.; Khademhosseini, A. Cell-Laden Microengineered and Mechanically Tunable Hybrid Hydrogels of Gelatin and Graphene Oxide. *Adv. Mater.* **2013**, *25* (44), 6385–6391. https://doi.org/10.1002/adma.201301082.
21. Shin, S. R.: Bae, H.; Cha, J. M.; Mun, J. Y.; Chen, Y.-C.; Tekin, H.; Shin, H.; Farshchi, S.; Dokmeci, M. R.; Tang, X.; Khademhosseini, A. Carbon Nanotube Reinforced Hybrid Microgels as Scaffold Materials for Cell Encapsulation, ACS Nano **2012** , 6 , 362 .
